# Impact of Underlying Liver Disease on the Risk and Prognostic Factors of Breast Cancer Liver Metastases: A Retrospective Multicenter Cohort Study

**DOI:** 10.1002/cam4.71761

**Published:** 2026-04-24

**Authors:** Xiaowen Wang, Yuan Zhang, Qiwei Yang, Luo Yang, Yanhui Wang, Xiangxin Zeng, Long Zou, Yujuan Guo, Wenyang Zhou, Yuqi Jiang, Jiezhong Wu, Peng Zhang, Song Zhou, Guangjuan Zheng, Tao Huang, Genshu Wang, Kunpeng Hu

**Affiliations:** ^1^ Department of Thyroid and Breast Surgery, the Third Affiliated Hospital of Sun Yat‐Sen University Guangzhou China; ^2^ Department of Pathology, Guangdong Provincial Hospital of Traditional Chinese Medicine Guangzhou China; ^3^ Department of Hepatic Surgery and Liver Transplantation, Guangdong Provincial Hospital of Traditional Chinese Medicine Guangzhou China; ^4^ Breast Cancer Center, Shandong Cancer Hospital and Institute, Shandong First Medical University, and Shandong Academy of Medical Sciences Jinan Shandong China; ^5^ Zhangzhou Municipal Hospital of Fujian Province Zhangzhou Fujian China; ^6^ The 909th Hospital School of Medicine, Xiamen University China

**Keywords:** breast cancer liver metastasis, HBsAg infection, multicenter cohort study, nonalcoholic fatty liver, propensity score matching

## Abstract

**Objective:**

To examine factors influencing breast cancer liver metastases (BCLM) and assess the impact of underlying liver diseases (nonalcoholic fatty liver and HBsAg infection) on BCLM development.

**Methods:**

Patients diagnosed with breast cancer at four affiliated hospitals in China between 2014 and 2024 were included. Logistic regression was used to identify factors associated with BCLM. Propensity score matching (PSM) and Kaplan–Meier analyses were performed to evaluate the prognostic impact of underlying liver diseases.

**Results:**

A total of 3653 breast cancer patients were included, among whom 387 (11%) were identified with liver metastasis (LM). Factors including nonalcoholic fatty liver (NAFL) and HBsAg (hepatitis B surface antigen) infection were independently associated with a lower risk of BCLM (NAFL: *p* < 0.001; HBsAg: *p* = 0.011). Subsequent analysis stratified by the severity of NAFL indicated that mild NAFL was associated with a lower risk of BCLM, whereas moderate‐to‐severe NAFL was associated with a higher risk of BCLM (*p* = 0.01 and *p* = 0.02, respectively). Survival analysis showed that HBsAg infection was associated with significantly longer liver metastasis‐free survival (LMS) and overall survival (OS) (both *p* < 0.01). Further survival analysis, stratified by the presence of NAFL, revealed that mild NAFL could prolong both LMS and OS, while moderate‐to‐severe NAFL not only shortened LMS, but also shortened OS after LM (OSLM), so that OS was significantly shortened *(p* < 0.01 for mild NAFL; *p* < 0.05 for LMS and *p* < 0.01 for OSLM and OS in moderate‐to‐severe NAFL). Furthermore, consistent results on OS and OSLM were obtained even after employing 1:1 PSM to account for other covariate interferences (both *p* < 0.01).

**Conclusion:**

Mild NAFL may be associated with reduced LM and improved prognosis, while moderate‐to‐severe NAFL appears to correlate with increased LM risk and worse clinical outcomes. Furthermore, HBsAg infection may be linked to suppressed LM and extended OS for patients with BCLM.

## 1 Introduction

Breast cancer (BC) ranks as the second most common cancer globally, following lung cancer, and is the fifth leading cause of cancer‐related deaths. It is the primary cause of cancer mortality among women. The global prevalence of BC surpasses that of all other cancers, affecting approximately 20% of the global population, with incidence rates on the rise [1, 2, 3]. At the time of diagnosis, approximately 5% to 15% of BC patients present with distant metastasis. Following standard treatment, 20% to 30% of BC patients experience recurrence or distant metastasis, commonly to the bone, lung, liver, or brain [4]. Metastatic breast cancer (MBC) is incurable, and almost all BC deaths are caused by distant metastases. Notably, as the third most common metastatic organ in breast cancer, patients with breast cancer liver metastases (BCLM) have a worse prognosis than those with bone, lung, or other organ metastases, with a 5‐year survival rate of only 3.8%–12% [5, 6]. Because all patients with breast cancer are at risk of developing liver metastasis, identifying risk factors associated with BCLM is essential for developing individualized treatment strategies and improving survival outcomes. As research on the risk factors of BCLM continues to expand, it has garnered attention in numerous single‐center, small‐sample retrospective studies. However, there remains a lack of large‐sample, multi‐center studies.

The presence of underlying liver disease significantly impacts the development and prognosis of metastatic liver cancer. Nonalcoholic fatty liver (NAFL) and HBsAg infection are prevalent underlying liver diseases that can alter liver structure and function, subsequently influencing the microenvironment for metastatic cancer cells to colonize and grow. Consequently, these conditions have garnered attention as key research areas in the investigation of risk factors for metastatic liver cancer in recent years [7, 8, 9]. NAFL is widely recognized as the most prevalent liver disorder globally, with a prevalence ranging from 6.3% to 33.0% [10]. The impact of NAFL on metastatic liver cancer has been a topic of debate within the scientific community. While certain studies propose that hepatic steatosis (HS) in its early stages may create a conducive microenvironment for the initiation and progression of colorectal cancer liver metastasis (CCLM), conflicting research suggests that HS may actually impede CCLM [11, 12]. Hamady et al.'s [13] analysis of experiments investigating the correlation between NAFL and primary and secondary hepatic malignant tumors suggests that NAFL is linked to a higher incidence of CCLM, but does not show a clear correlation with primary hepatocellular carcinoma incidence. BC patients are often accompanied by NAFL, and there are few studies on the relationship between NAFL and BCLM. Ocak et al. [14] found a higher prevalence of patients with NAFL in BCLM; however, the study had limitations such as a small sample size and weak evidence. In contrast, a two‐center cohort study conducted by Wu et al. [15] indicated that NAFL may independently reduce LM in BC patients. The impact of NAFL on BCLM requires further investigation, but these findings hold significant implications for future clinical practice.

The prevalence of HBsAg in China is notably elevated, with infection rates ranging from 5% to 6% in the general population. HBsAg infection is established as a significant risk factor for primary hepatocellular carcinoma, with approximately 83.77% of cases attributed to this infection [16, 17]. Nevertheless, the relationship between HBsAg infection and metastatic liver cancer is not fully understood, and remains a topic of debate similar to the controversial role of NAFL in metastatic liver cancer. Several studies have proposed HBsAg as a potential protective factor for metastatic liver cancer, highlighting its ability to bolster liver immunity and induce tumor cell apoptosis [18, 19]. Conversely, conflicting evidence exists regarding this assertion [20, 21]. Nonetheless, there is a paucity of research examining the impact of HBsAg infection on BCLM.

This study gathered data from four medical centers on BCLM patients, with a specific focus on analyzing the influence of underlying liver diseases on the progression and prognosis of BCLM. The findings offer valuable data to support clinical diagnosis and treatment of BCLM, as well as provide a direction for future research on the molecular mechanisms involved.

## 2 Materials and Methods

### 2.1 Ethics

Our study was approved by the ethics committees of the Third Affiliated Hospital of Sun Yat‐sen University, Guangdong Provincial Hospital of Chinese Medicine, Shandong Provincial Qianfoshan Hospital, and Zhangzhou Affiliated Hospital of Fujian Medical University and complied with the Declaration of Helsinki.

### 2.2 Study Population

The information of all patients diagnosed with BC in the Third Affiliated Hospital of Sun Yat‐sen University, Guangdong Provincial Hospital of Chinese Medicine, Shandong Provincial Qianfoshan Hospital, and Zhangzhou Affiliated Hospital of Fujian Medical University from May 2014 to May 2024 was collected. This patient information was sourced from the Hospital Information System (HIS) and encompassed basic patient details, treatment records, pathological characteristics, and follow‐up prognoses. **Inclusion criteria:** Patients with BC confirmed by histopathological examination; Patients with BCLM confirmed by imaging examination and liver biopsy; Patients who underwent abdominal (liver) ultrasonography and testing for HBsAg within 30 days before or after the diagnosis of primary breast cancer were included. NAFL status was assessed at the time of initial breast cancer diagnosis and prior to the occurrence of liver metastasis; Patients with BC and BCLM with complete medical records.

**Exclusion criteria:** Patients with breast cancer diagnosed with distant metastasis at initial presentation; patients with a history of other malignant tumors; patients with a documented history of chronic alcohol consumption; patients with hepatitis C virus infection, autoimmune liver disease, or clinically documented liver cirrhosis based on available medical records; and patients with incomplete medical records or a follow‐up duration of less than 30 days.

### 2.3 Follow‐Up

The follow‐up period commenced upon the pathological diagnosis of BC patients. Follow‐up methods encompassed the examination of inpatient and outpatient records in the HIS, telephone follow‐up, and verification of survival status through the public security department. Data on variables including age at onset, body mass index (BMI), menstrual status, history of underlying liver diseases, primary tumor site, neoadjuvant therapy (NAT), surgical approach for the primary tumor, pathological subtype of the primary tumor, vascular invasion, histological grade, molecular subtyping, and tumor staging were collected. Additionally, 5‐year liver metastasis‐free survival (LMS), overall survival (OS), and overall survival after liver metastasis (OSLM) were also documented.

## 3 Statistics

Univariable and multivariable logistic regression analyses were conducted to examine factors associated with the risk of developing breast cancer liver metastasis (BCLM). Propensity score matching (PSM) analysis was employed to mitigate potential biases arising from differences in covariate distribution between patients with and without underlying liver disease. Nearest‐neighbor matching with a caliper width of 0.02 was used to achieve a 1:1 matching ratio. LMS, OS, and OSLM were estimated using the Kaplan–Meier method, and 5‐year survival rates were derived from the corresponding survival curves. Cox proportional hazards regression models were used to evaluate the associations between underlying liver diseases (NAFL severity and HBsAg status) and survival outcomes (OS and OSLM), where applicable. The proportional hazards assumption was evaluated using time‐dependent covariate analyses by including interaction terms between time and key covariates in the Cox regression models. Data analysis was conducted using SPSS version 25.0 (IBM, Armonk, NY, USA), with statistical significance defined as *p* < 0.05.

## 4 Results

### 4.1 Baseline Characteristics

**FIGURE 1:**
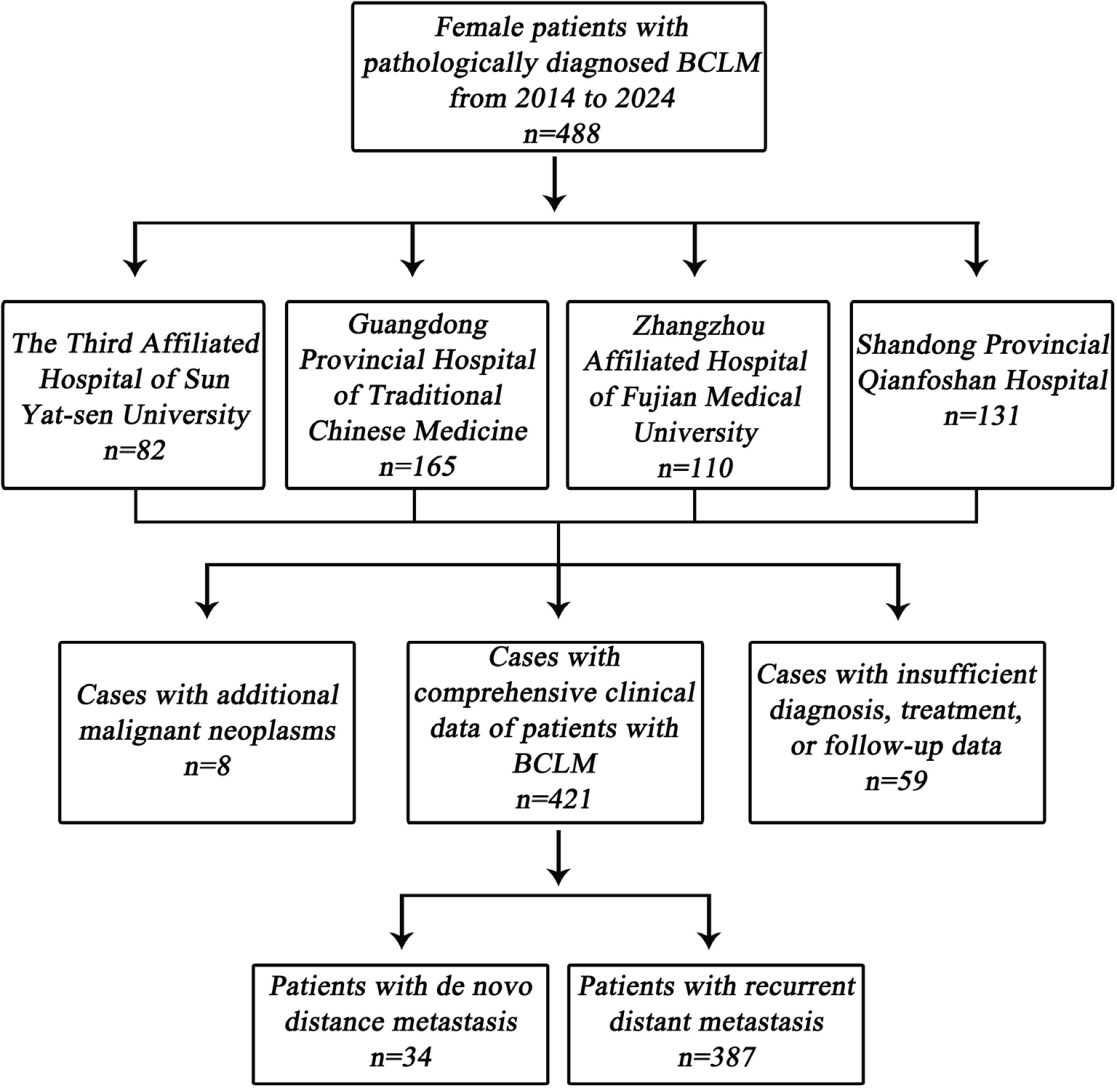
Screening flowchart of research objects. BCLM, breast cancer liver metastases.

This study included 3653 eligible patients with BC from four medical centers between 2014 and 2024, including 3266 patients without LM and 387 patients with LM (a rate of 11%) (Figure 1). Baseline characteristics indicated a lower prevalence of underlying liver diseases in the cohort with LM compared to the cohort without LM. The incidence of NAFL was 20% vs. 36%, and the incidence of HBsAg infection was 8% vs. 13%. The baseline data also revealed that radical mastectomy was the predominant surgical approach among patients with BC, with breast‐conserving surgery being less commonly employed. Unilateral primary tumors were more prevalent compared to bilateral cases, and the utilization of pre‐operative NAT was relatively infrequent. Ductal carcinoma in situ represented a smaller proportion of cases, whereas invasive BC of no special type constituted a higher relative proportion. Additionally, the luminal A molecular subtype was more frequently observed, whereas the triple‐negative subtype was less common (Table 1).

**TABLE 1:** Baseline characteristics of BC patients with/without LM.

Characteristic	Without LM group (*n* = 3266, 89%)	With LM group (*n* = 387, 11%)	*P*‐value
Age (yr)			
< 50	1,508 (46%)	175 (45%)	*p* = 0.722
≥ 50	1,758 (54%)	212 (55%)	
BMI (kg/m2)			
< 24	2,090 (64%)	267 (69%)	*p* = 0.052
≥ 24	1,176 (36%)	120 (31%)	
Menstrual status			
Premenopausal	1,805 (55%)	211 (55%)	*p* = 0.781
Postmenopausal	1,461 (45%)	176 (45%)	
NAFL			
None	2,067 (63%)	308 (80%)	*P* < 0.001
Mild	796 (24%)	40 (10%)	
Moderate‐to‐severe	403 (12%)	39 (10%)	
HBsAg infection			
Negative	2,848 (87%)	355 (92%)	*P* = 0.011
Positive	418 (13%)	32 (8%)	
NAT			
No	2,082 (64%)	263 (68%)	*p* = 0.103
Yes	1,184 (36%)	124 (32%)	
Surgical approach			
Radical mastectomy	2,238 (69%)	267 (69%)	*p* = 0.105
Breast‐Conserving	842 (26%)	90 (23%)	
Others	186 (5%)	30 (8%)	
Primary lesion site			
Left	1,567 (48%)	188 (49%)	*p* = 0.914
Right	1,526 (47%)	179 (46%)	
Bilateral	173 (5%)	20 (5%)	
T category			
T1/T2	2,906 (89%)	334 (86%)	*p* = 0.117
T3/T4	360 (11%)	53 (14%)	
N category			
N0	1,424 (44%)	148 (38%)	*p* = 0.044
N1–N3	1,842 (56%)	239 (62%)	
Vascular invasion			
No	466 (14%)	72 (19%)	*p* = 0.023
Yes	2,800 (86%)	315 (71%)	
Pathological subtype			
DCIS	294 (9%)	30 (8%)	*P* < 0.001
IBC‐NST	2,565 (79%)	299 (77%)	
IBC	407 (12%)	58 (15%)	
Histological grade			
I/II	2,320 (71%)	250 (65%)	*p* = 0.009
III	946 (29%)	137 (35%)	
Molecular subtyping			
Luminal‐A	1,894 (58%)	196 (51%)	*p* = 0.006
Luminal‐B	490 (15%)	74 (19%)	
HER2‐OE	555 (17%)	58 (15%)	
TNBC	327 (10%)	59 (15%)	

### 4.2 Analysis of Factors Influencing BCLM

The univariate analysis revealed that several factors, including age, menstrual status, BMI, NAT, surgical approach, primary tumor site, T tumor staging, and pathological subtype, did not exhibit significant differences between the BC with LM group and the BC without LM group (*p* > 0.05). Conversely, vascular invasion (OR 1.37, 95% CI 1.04–1.18, *p* = 0.02), molecular subtyping (OR 1.14, 95% CI 1.04–1.26, *p* = 0.01), histological grade (OR 1.34, 95% CI 1.08–1.68, *p* = 0.01), and N tumor staging (OR 1.25, 95% CI 1.01–1.55, *p* = 0.04) were identified as risk factors for BCLM, while NAFL (OR 0.64, 95% CI 0.54–0.77; *p* < 0.01) and HBsAg infection were associated with a lower risk of liver metastasis (OR 0.61, 95% CI 0.42–0.89; *p* = 0.01) and were found to be protective factors against BCLM. Notably, further stratification of NAFL indicated that mild NAFL is a protective factor for BCLM (OR 0.52, 95% CI 0.33–0.82, *p* = 0.01), whereas moderate‐to‐severe NAFL serves as a risk factor against it (OR 1.51, 95% CI 1.09–2.19; *p* = 0.01). A further multivariate logistic regression model was utilized to examine the variables linked to BCLM. In agreement with the results of the univariate analysis, NAFL and HBsAg infection were determined to be independent protective factors against BCLM. Notably, the stratification of NAFL demonstrated that mild NAFL was associated with a lower risk of liver metastasis, whereas moderate‐to‐severe NAFL was associated with an increased risk. Furthermore, vascular invasion, histological grade, and molecular subtyping were identified as independent risk factors for BCLM (Table 2).

**TABLE 2:** Logistic regression of factors associated with BCLM.

Parameter	Univariate analysis		Multivariate analysis	
	OR (95% CI)	*P*‐value	OR (95% CI)	*P*‐value
N category	1.25 (1.01–1.55)	0.04	1.40 (1.10–1.77)	0.01
Vascular invasion	1.37 (1.04–1.81)	0.02	2.18 (1.45–3.28)	< 0.01
Histological grade	1.34 (1.08–1.68)	0.01	1.05 (0.74–1.50)	0.78
Molecular subtyping	1.14 (1.04–1.26)	0.01	1.14 (1.00–1.29)	0.04
HBsAg infection	0.61 (0.42–0.89)	0.01	0.25 (0.16–0.41)	< 0.01
NAFL	0.64 (0.54–0.77)	< 0.01	0.65 (0.54–0.78)	< 0.01
None				
Mild	0.52 (0.33–0.82)	0.01	0.47 (0.29–0.75)	0.01
Moderate to sereve	1.51 (1.09–2.19)	0.02	1.44 (1.00–2.08)	0.04

**FIGURE 2:**
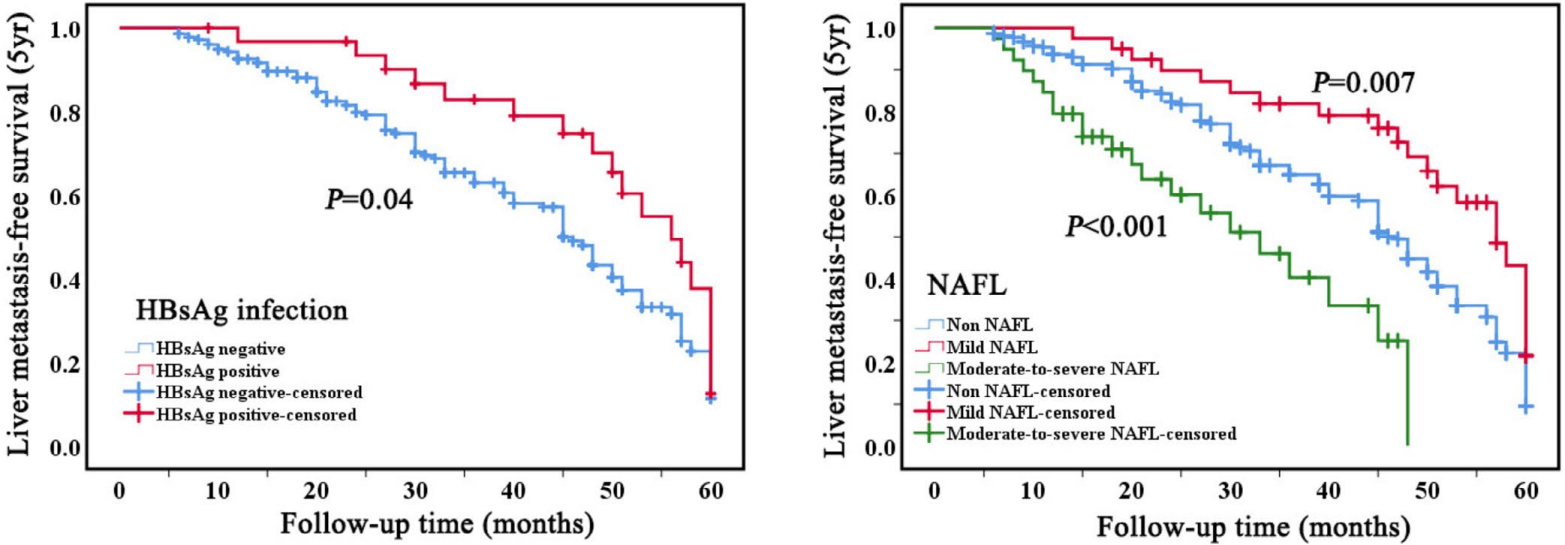
Kaplan–Meier curves depicting 5‐year metastasis‐free survival for patients with breast cancer liver metastases, stratified by NAFL (right) and HBsAg infection (left). The *P*‐value was calculated using the unadjusted log‐rank test. Five‐year, NAFL, nonalcoholic fatty liver.

Further analysis was conducted within the BCLM group to evaluate the influence of underlying liver diseases on the 5‐year LMS. The Kaplan–Meier analysis indicated that HBsAg infection extended the LMS. In the case of NAFL, mild NAFL was associated with a prolonged LMS, whereas moderate‐to‐severe NAFL significantly reduced LMS (Figure 2).

### 4.3 Survival and Prognostic Analysis

**FIGURE 3:**
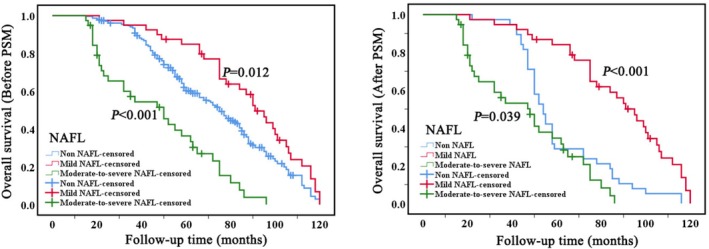
Kaplan–Meier survival curves depicting overall survival for patients with breast cancer liver metastases, stratified by NAFL, before (left) and after (right) PSM. *P*‐value from the log‐rank test. PSM, propensity score matching; NAFL, nonalcoholic fatty liver.

**FIGURE 4:**
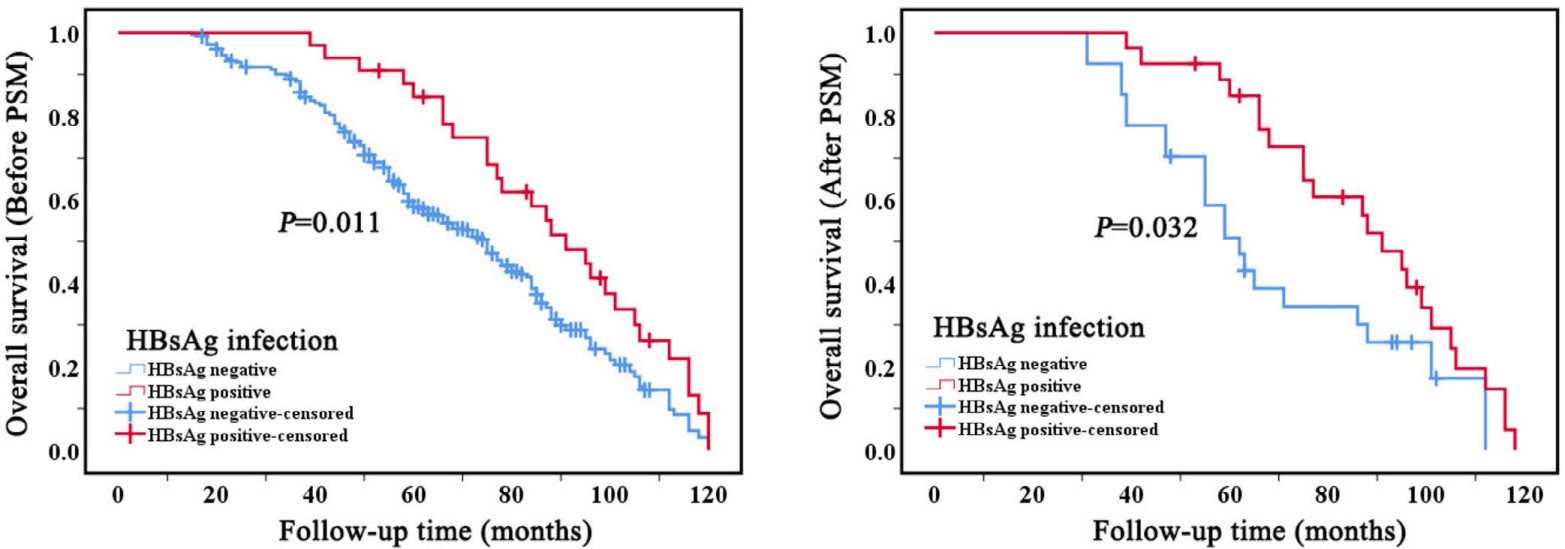
Kaplan–Meier survival curves depicting overall survival for patients with breast cancer liver metastases, stratified by HBsAg infection, before (left) and after (right) PSM. *P*‐value from the log‐rank test. PSM, propensity score matching.

The median survival time for 387 patients with BCLM was 75 months (SD: 2.7, 95% CI: 68.9–81.0). NAFL significantly influences the OS of patients with BCLM at a statistically significant level (*p* < 0.001). Compared to the non‐NAFL group, patients with mild NAFL exhibit a longer OS, with a median OS of 91 months (SD: 4.9, 95% CI: 81.2–100.7), while those with moderate‐to‐severe NAFL show a shorter OS, with a median OS of 50 months (SD: 9.5, 95% CI: 31.2–68.7). The corresponding mean and median survival estimates with 95% confidence intervals are summarized in Supplementary Table 7. Consistent associations between NAFL severity, HBsAg status, and overall survival were observed in Cox proportional hazards models, supporting the findings of the Kaplan–Meier analyses. To control for potential baseline confounding factors, a 1:1 PSM was performed among the three groups, yielding 38 matched patients per group (Supplementary Table 1). Post‐matching analysis indicated that the influence of NAFL on OS persisted in alignment with the pre‐matching findings (Figure 3, Supplementary Table 2). Additionally, HBsAg infection also significantly impacts the OS of patients with BCLM (*p* = 0.011). The HBsAg‐positive group demonstrates a relatively longer OS compared to the HBsAg‐negative group, with a median OS of 91 months (SD: 7.2, 95% CI: 76.7–105.2). To eliminate the impact of other factors between the HBsAg‐positive and negative groups, patients from each group were matched 1:1 using PSM, yielding 27 matched patients per group (Supplementary Table 3). Post‐matching analysis indicated that the influence of HBsAg infection on OS persisted in alignment with the pre‐matching findings (Figure 4, supplementary Table 4).

**FIGURE 5:**
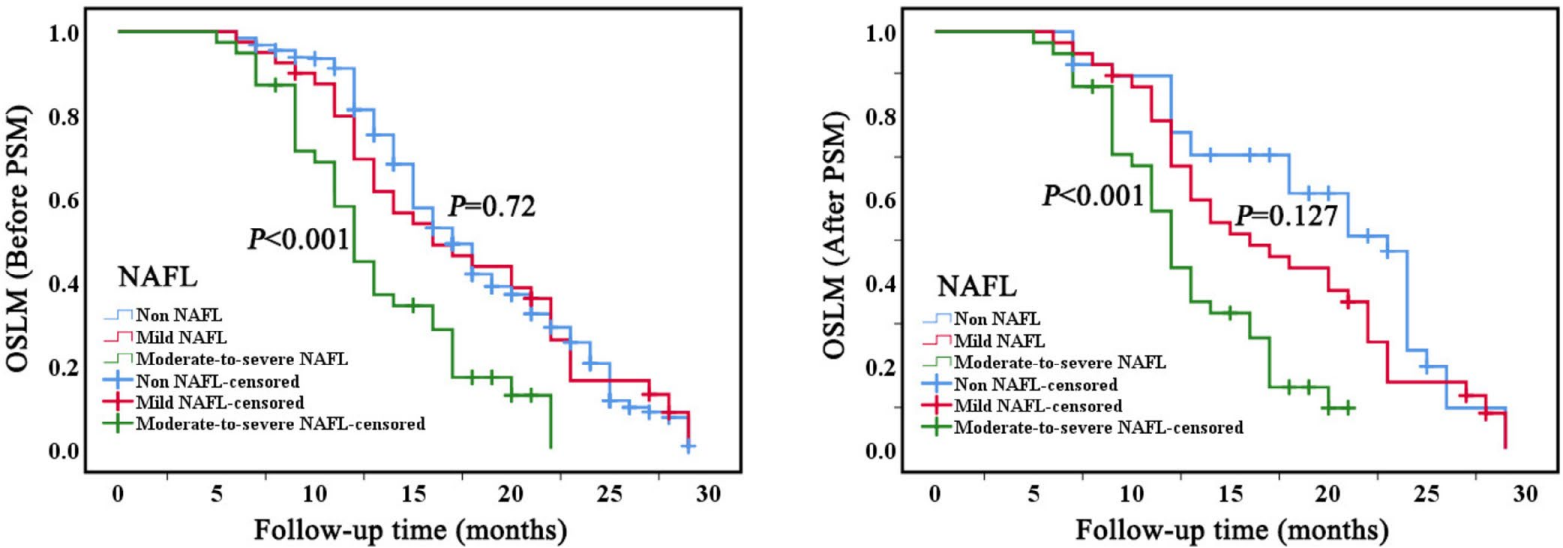
Kaplan–Meier survival curves depicting OSLM for patients with breast cancer liver metastases, stratified by NAFL, before (left) and after (right) PSM. *P*‐value from the log‐rank test. PSM, propensity score matching; NAFL, nonalcoholic fatty liver; OSLM, overall survival after liver metastases.

**FIGURE 6:**
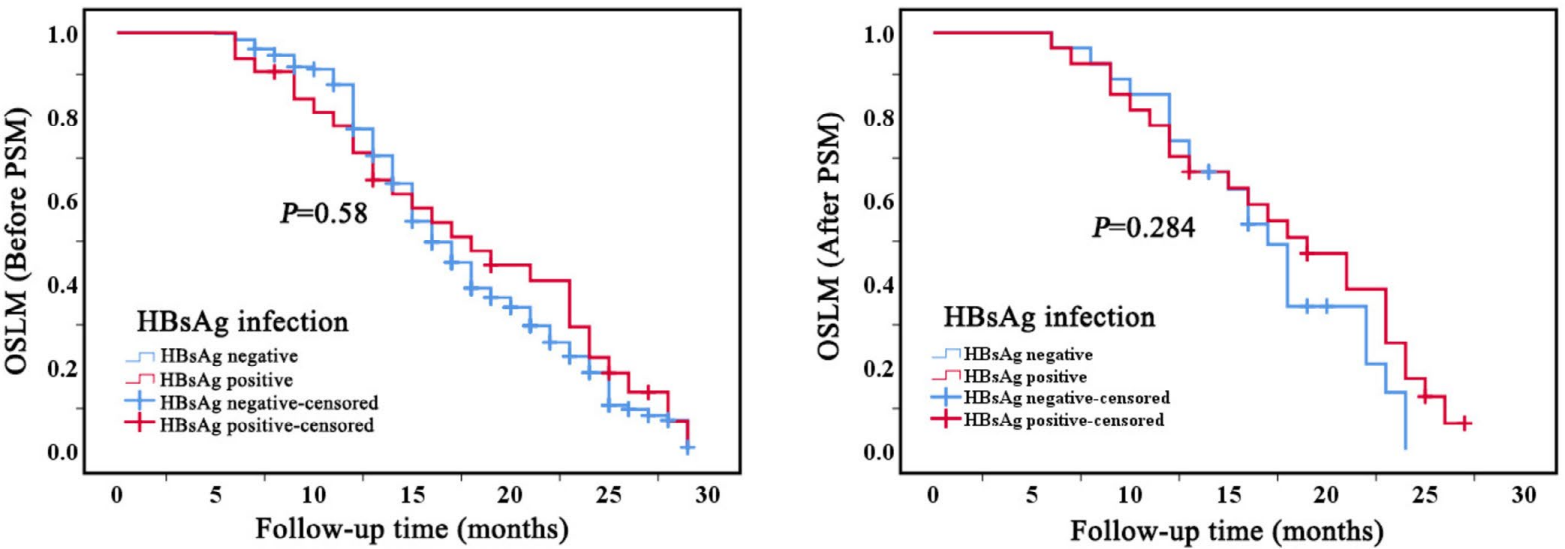
Kaplan–Meier survival curves depicting OSLM for patients with breast cancer liver metastases, stratified by HBsAg infection, before (left) and after (right) PSM. *P*‐value from the log‐rank test. PSM, propensity score matching; OSLM, overall survival after liver metastases.

The median survival time after LM for 387 patients with BCLM was 17 months (SD: 0.5, 95% CI: 16.0–17.9). NAFL significantly influences the OSLM of patients with BCLM (*p* < 0.001). Mild NAFL demonstrates no significant variance compared to those with non‐NAFL, exhibiting a median OSLM of 16 months (SD:2.4, 95% CI:11.1–20.8). Conversely, patients with moderate‐to‐severe NAFL exhibit a significantly shorter OSLM, with a median of 12 months (SD:0.6, 95% CI:10.8–13.1). Detailed mean and median OSLM estimates are provided in Supplementary Table 7. Cox proportional hazards analyses yielded results consistent with the Kaplan–Meier estimates, and no violation of the proportional hazards assumption was detected. The results remained consistent even after the application of PSM (Figure 5, supplementary Table 5). Additionally, the presence of HBsAg positivity does not significantly impact OSLM, with a median survival time of 18 months (SD:2.6, 95% CI:12.7–23.2) in comparison with HBsAg‐negative individuals. Furthermore, consistent results were obtained even after employing 1:1 PSM to account for other covariate interferences (Figure 6, supplementary Table 6).

## 5 Discussion

BC has the potential to metastasize to distant sites, even in its early stages, with a higher incidence of metastasis observed in advanced cases. The bone, lung, liver, and brain are the most frequently affected sites of metastasis. The liver is the exclusive site of distant metastasis in BC, accounting for about 5%–12%, with isolated liver lesions present in only 4%–5% of patients with BCLM [22, 23]. Although liver involvement is not the most prevalent site of BC metastasis, it serves as an independent prognostic indicator of inferior OS when compared to other sites, such as bone metastasis. Patients diagnosed with BCLM often face a poor prognosis, as evidenced by a median OS of approximately 4–8 months in the absence of treatment. Even among individuals who exhibit a positive response to systemic therapy, the median OS following diagnosis of BCLM is limited to 18–24 months [24]. Despite initial stability in treatment response lasting 1–2 years, disease progression is a common occurrence. The 5 and 10‐year OS rates following diagnosis are notably low, at around 27% and 13%, respectively [25]. This study analyzed data from patients with BC at four medical centers. The results indicated that the occurrence of isolated LM in patients with BC was consistent with findings from Wang's research, approximately 11%. The median OSLM was 17 months, and the median OS was approximately 75 months.

BCLM is a complex process involving local infiltration of BC cells, dissemination into the circulatory system, migration to target organs through the circulation, adhesion and colonization at the target site, and formation of metastatic foci. This process is influenced by various factors, including the environmental characteristics of BC itself, multiple chemotactic factors during the metastatic process, and the hepatic microenvironment [26, 27, 28]. The hepatic microenvironment and structure of the liver sinus are known to exert a significant influence on the progression or regression of BCLM, yet the underlying biological mechanisms driving this phenomenon remain incompletely understood. NAFL is a clinical condition stemming from a pathological syndrome marked by widespread accumulation of fat in liver cells and impaired lipid processing, independent of alcohol consumption and other known liver‐damaging agents. The influence of NAFL on metastatic liver cancer has sparked considerable scholarly discussion. Some scholars argue that NAFL‐induced disruptions in lipid metabolism, excessive oxidative stress, and inflammatory responses lead to hepatocellular damage or apoptosis, creating a favorable environment for the metastasis of cancer cells [29, 30]. However, the prevailing view among most scholars is that NAFL‐induced alterations in the hepatic microenvironment inhibit the LM of cancer cells. HS leads to progressive distortion and obstruction of the hepatic vasculature, causing disruption of the native vascular architecture and hindering blood flow. This impediment hinders the efficient transport of cancer cells to the liver, thereby decreasing their chances of engraftment in the liver [31]. Furthermore, NAFL may inhibit LM by suppressing hepatic angiogenesis and pyrimidine nucleoside phosphorylase activity [32]. Certain perspectives propose that the accumulation of fat in the liver can influence the immune microenvironment, leading to the suppression of LM. Specifically, the buildup of fat in the liver activates Kupffer cells, allowing them to capture and eradicate circulating cancer cells, hindering their proliferation within the liver, and enhancing the secretion of IFN‐γ and other cytokines, contributing to a synergistic anti‐cancer response that ultimately diminishes the implantation of cancer cells [33]. Therefore, despite the considerable advancements in the study of NAFL influence on LM, the definitive impact of NAFL on either inhibiting or promoting LM of extrahepatic tumors remains uncertain. As a result, there is a current deficiency in effective strategies for the prevention and treatment of metastatic liver cancer.

Research on the impact of NAFL on BCLM typically categorizes it into non‐NAFL and NAFL based solely on the presence or absence of hepatic fat deposition, without further stratifying them according to the degree and extent of NAFL involvement into mild, moderate, and severe categories [15, 30, 34]. However, the impact on the structure and function of the liver caused by varying degrees of NAFL is quite different. As a result, we hypothesize that varying degrees of NAFL may exert differential effects on BCLM. This study involves the collection and classification of patient data into three distinct categories based on hepatic ultrasound findings: Non‐NAFL, mild‐NAFL, and moderate‐to‐severe NAFL. The results indicate that mild NAFL was associated with a lower incidence of liver metastasis, whereas moderate‐to‐severe NAFL was associated with a higher incidence of liver metastasis. One possible explanation is that mild NAFL may be accompanied by subtle alterations in liver tissue structure and modulation of the hepatic immune microenvironment, which could potentially reduce the implantation or growth of metastatic cancer cells. In contrast, progression from moderate to severe NAFL is often accompanied by more pronounced metabolic dysregulation, oxidative stress, and chronic inflammation. These pathological changes may contribute to hepatocellular injury and create a hepatic microenvironment that is potentially more permissive to the establishment of BCLM. However, it is imperative to conduct further multi‐center, large‐scale clinical studies to provide empirical evidence regarding the impact of fatty liver on the development of BCLM. Additionally, fundamental research is required to establish a theoretical foundation for this relationship. Furthermore, our results are consistent with previous studies suggesting an association between HBsAg infection and a lower incidence of liver metastasis in breast cancer [35, 36]. Nonetheless, the specific molecular pathways through which HBsAg infection exerts this effect remain poorly understood. Some studies suggest that HBV replication may bolster the immune response against metastatic cancer cells by increasing the cytotoxicity of T lymphocytes and Kupffer cells, as well as activating cytokines like TNF‐α and IFN‐γ, thus enhancing anti‐tumor activity [9, 18]. These hypotheses are speculative and are not supported by direct mechanistic or molecular evidence in the present study; therefore, additional experimental investigations are required to fully elucidate the underlying mechanisms.

Our study is also subject to certain limitations. It is a multicenter retrospective analysis, and variations in the diagnosis and management of BCLM across different medical institutions may exist. Additionally, this study encompasses case data from the past decade, and with advancements in technology and treatment paradigms, updates in the approach to diagnosing and treating BCLM may have occurred. These factors could introduce certain selection biases. Furthermore, potential interference among various single factors may introduce certain biases to the results. We attempted to mitigate this confounding interference by employing PSM to control for covariates.

In addition, nonalcoholic fatty liver was assessed using abdominal ultrasonography, which is a cost‐effective and widely used screening modality in clinical practice. However, ultrasonography has limited sensitivity for detecting mild steatosis and does not allow precise quantification of hepatic fat content. Therefore, misclassification of NAFL severity cannot be completely excluded. Although patients with other major chronic liver diseases, such as hepatitis C virus infection, autoimmune liver disease, and clinically documented cirrhosis, were excluded to reduce potential interference, residual confounding related to unmeasured or incompletely recorded liver conditions may still exist. Furthermore, detailed information on systemic treatments, including chemotherapy, endocrine therapy, and HER2‐targeted therapy, was not comprehensively available and could not be fully adjusted for, which may have contributed to residual confounding. Moreover, the relatively small number of patients with mild and moderate‐to‐severe NAFL within the liver metastasis cohort may have limited statistical power and reduced the robustness of subgroup analyses. Although HBsAg infection appeared to be associated with favorable outcomes, competing risks, such as liver‐related mortality unrelated to cancer progression, were not formally evaluated, which limits causal interpretation of this finding. Therefore, larger prospective studies with more comprehensive imaging assessments, detailed treatment data, and appropriate competing risk analyses are warranted to validate and extend our findings.

In summary, our comprehensive retrospective cohort study, conducted across multiple centers with a large sample size, has demonstrated the influence of underlying liver diseases on BCLM and their implications for patient prognosis. Although we have not delved into the specific molecular mechanisms underlying this impact, the findings of our study provide valuable clinical data to support the diagnosis and treatment of BCLM. Furthermore, our research offers important insights for guiding future investigations into the molecular mechanisms involved in this area.

## Author Contributions

**Yanhui Wang:** formal analysis, supervision, investigation. **Xiaowen Wang:** writing – original draft, writing – review and editing, conceptualization, investigation, validation, methodology, software, data curation, supervision. **Luo Yang:** investigation, validation. **Xiangxin Zeng:** software, investigation. **Long Zou:** software, investigation. **Peng Zhang:** methodology, software, investigation. **Yujuan Guo:** software, data curation, investigation. **Yuqi Jiang:** methodology, software, investigation. **Qiwei Yang:** writing – review and editing, investigation, data curation, formal analysis. **Guangjuan Zheng:** software, investigation. **Wenyang Zhou:** software, data curation, investigation. **Tao Huang:** investigation. **Song Zhou:** methodology, software, investigation. **Kunpeng Hu:** conceptualization, writing – review and editing, methodology, formal analysis, supervision, data curation. **Jiezhong Wu:** methodology, software, investigation. **Genshu Wang:** investigation, writing – review and editing, project administration, supervision, resources, funding acquisition.

## Funding

This work was supported by the National Natural Science Foundation of China (Grant Nos. P02565, 82070673, 82370663, 82100691, 82270688).

## Conflicts of Interest

The authors declare no conflicts of interest.

## Supporting information


**Supplementary Table 1** Baseline characteristics of patients with BCLM stratified by NAFL severity before and after PSM.

**Supplementary Table 2** Mean and median overall survival (OS) according to NAFL severity before and after PSM.

**Supplementary Table 3** Baseline characteristics of patients with BCLM stratified by HBsAg status before and after PSM.

**Supplementary Table 4** Mean and median overall survival (OS) according to HBsAg status before and after PSM.

**Supplementary Table 5** Mean and median overall survival after liver metastasis (OSLM) according to NAFL severity before and after PSM.

**Supplementary Table 6** Mean and median overall survival after liver metastasis (OSLM) according to HBsAg status before and after PSM.

**Supplementary Table 7** Kaplan–Meier estimates of OS and OSLM stratified by NAFL severity and HBsAg status, with and without PSM.

## Data Availability

The data that support the findings of this study are available from the corresponding author upon reasonable request.
